# Supplementary material for: Early Treatment Outcomes for Bloodstream Infections Caused by Potential AmpC Beta-Lactamase-Producing Enterobacterales with Focus on Piperacillin/Tazobactam: A Retrospective Cohort Study
Source: Antibiotics (Basel). 2021 Jun 2;10(6):665. doi: 10.3390/antibiotics10060665 (PMC8229083; doi:10.3390/antibiotics10060665)
Supplement: Supplementary file 1 [file antibiotics-10-00665-s001.zip › antibiotics-1230165-supplementary.pdf]

antibiotics

MDPI

Online Supplemental material to:

Early Treatment Outcomes for Bloodstream Infections Caused by Potential AmpC Beta-Lactamase-Producing Enterobacterales with Focus on Piperacillin/Tazobactam: A Retrospective Cohort Study

Lena Herrmann [1], Aurelia Kimmig [1], Jürgen Rödel [2], Stefan Hagel [1], Norman Rose [1,3], Mathias W. Pletz [1,†] and Christina Bahrs [1,4,*,†]

[1] Institute of Infectious Diseases and Infection Control, Jena University Hospital/Friedrich-Schiller-University, 07747 Jena, Germany; lena.herrmann@uni-jena.de (L.H.); aurelia.kimmig@med.uni-jena.de (A.K.); stefan.hagel@med.uni-jena.de (S.H.); norman.rose@med.uni-jena.de (N.R.); mathias.pletz@med.uni-jena.de (M.W.P.); christina.bahrs@med.uni-jena.de (C.B.)
[2] Institute of Medical Microbiology, Jena University Hospital/Friedrich-Schiller-University, 07747 Jena, Germany; Juergen.Roedel@med.uni-jena.de
[3] Clinic for Anaesthesiology and Intensive Care Medicine, Jena University Hospital/Friedrich-Schiller-University, 07747 Jena, Germany
[4] Department of Medicine I, Division of Infectious Diseases and Tropical Medicine, Medical University of Vienna, 1090 Vienna, Austria
* Correspondence: christina.bahrs@med.uni-jena.de
† These authors contributed equally to this work.

Adjusted comparison of early treatment response between Carbapenem and Piperacillin/Tazobactam regimen

As shown in Table 1, there is evidence that the groups of patients defined by the empiric treatment regimens differ regarding patient characteristics, comorbidities, disease severity and bacteremia sources. This may limit the interpretation of mean differences in the early treatment response between the empiric Carbapenem and Piperacillin/Tazobactam regimen. The fairly low sample size of n = 163 cases does not allow for complex statistical models to adjust for distributional differences in covariates across treatment groups. Therefore, we used a stepwise procedure. In a first step, the effect size of the covariate imbalance between the Carbapenem and Piperacillin/Tazobactam group was assessed using the absolute standardized mean differences (ASMD). ASMD < 0.2 for all potential covariates indicates good balancing of covariates across groups [1]. In a second step, all fully observed covariate (i.e., without missing values) and an ASMD ≥ 0.2 were included as covariates in a multiple logistic regression to estimate an adjusted treatment effect of the Carbapenem compared to the Piperacillin/Tazobactam regimen based on the full sample (Model A). Finally, the multiple logistic regression was extended by including the remaining incomplete covariates (i.e., covariates with missing values) with an ASMD ≥ 0.2 (Model B). In addition to the adjusted odds ratios, we report the average marginal effects (AVE), which are the adjusted differences in the percentage of cases with early treatment response between the Carbapenem group compared to the Piperacillin/Tazobactam group [2].

The ASMDs in Table S1 indicate small to moderate imbalance between the Carbapenem and the Piperacillin/Tazobactam group regarding some baseline characteristics, comorbidities, causative pathogens, and sources of BSI.

Table S1. Means (M), standard deviations (SD), numbers of missing values (Missing), and absolute standardized mean differences (ASMD) of baseline characteristics, comorbidities, disease severities and bacteremia sources of patients with SPICE bloodstream infection (BSI) treated with Carbapenem versus Piperacillin/Tazobactam.

Covariate	M (SD)	M (SD)	Missing	ASMD
Age	65.22 (16.36)	64.21 (12.39)	0	0.07 [-0.24, 0.38]
Body mass index	27.83 (8.42)	29.01 (17.53)	1	0.09 [-0.22, 0.39]
Charlson score	3.09 (2.04)	3.26 (2.23)	0	0.08 [-0.23, 0.39]
Pitt bacteremia score	1.86 (2.91)	2.55 (3.2)	0	0.22 [-0.08, 0.53]
SOFA score at baseline	4.71 (4.44)	6.22 (5.63)	6	0.30 [-0.02, 0.61]
Male sex	0.64 (0.23)	0.76 (0.18)	0	0.25 [-0.06, 0.56]
Implanted device	0.16 (0.13)	0.26 (0.19)	0	0.24 [-0.07, 0.55]
Comorbidities[2]				
Heart failure	0.31 (0.21)	0.32 (0.22)	0	0.02 [-0.29, 0.33]
Lung disease	0.25 (0.19)	0.32 (0.22)	0	0.16 [-0.15, 0.46]
Kidney disease	0.2 (0,16)	0.13 (0.12)	0	0.17 [-0.14, 0.48]
Liver disease	0.16 (0.13)	0.17 (0.14)	0	0.03 [-0.28, 0.34]
Diabetes	0.22 (0.17)	0.29 (0.21)	0	0.16 [-0.15, 0.47]
Metastatic carcinoma /leukemia	0.1 (0.09)	0.05 (0.05)	2	0.20 [-0.11, 0.51]
Causative pathogen[2]				
Enterobacter	0.53 (0.25)	0.54 (0.25)	0	0.01 [-0.3, 0.32]
Serratia	0.22 (0.17)	0.32 (0.22)	0	0.22 [-0.09, 0.52]
Other SPICE	0.25 (0.19)	0.17 (0.14)	0	0.19 [-0.12, 0.50]
Polymicrobial BSI	0.27 (0.20)	0.16 (0.13)	0	0.28 [-0.03, 0.59]
Main source of BSI[2]				
Unknown	0.25 (0.19)	0.2 (0.16)	0	0.13 [-0.18, 0.43]
Respiratory tract	0.24 (0.18)	0.35 (0.23)	0	0.26 [-0.05, 0.57]
Urinary tract	0.09 (0.08)	0.2 (0.16)	0	0.32 [0.01, 0.63]
Biliary tract	0.15 (0.13)	0.06 (0.06)	0	0.29 [-0.02, 0.60]
Vascular catheter	0.14 (0.12)	0.12 (0.11)	0	0.04 [-0.27, 0.35]
Intra-abdominal	0.05 (0.05)	0.07 (0.07)	0	0.10 [-0.21, 0.41]
Surgical site	0.11 (0.1)	0.12 (0.11)	0	0.03 [-0.27, 0.34]
Severe Immunodeficiency	0.11 (0.1)	0.11 (0.1)	7	0.01 [-0.30, 0.32]

[1]Implanted devices include cardiac and orthopedic implanted devices. [2]More than 1 answer is possible.

The estimated coefficients of the multiple logistic regression models A and B are shown in Table S2. The adjusted odds ratio of the Carbapenem regimen compared to the piperacillin/tazobactam regimen was 9.26, 95%CI = [3.78, 25.01], in Model A and 10.41, 95%CI = [3.97, 30.59], in Model B, which is statistically significant ($p < 0.001$). The adjusted odds ratios for the Carbapenem versus piperacillin/tazobactam regimen were even larger than the unadjusted odds ratio (3.59, 95%-CI = [1.83, 7.27]). The expected proportion of

cases with early treatment response in Model A was 53.2% under the Carbapenem regimen versus 18.5% under the piperacillin/tazobactam regimen. The estimates of Model B were nearly identical (53.2% under the Carbapenem regimen, 19.1% under piperacillin/tazobactam regimen). The estimated average marginal effects (AVE) were 32.4%, 95%CI = [22.7%, 42.0%] for Model A, and 32.1%, 95%CI = [22.2%, 42.7%] for Model B. Hence, crude and adjusted comparisons indicate a higher probability of an early treatment response under the Carbapenem regimen compared to the piperacillin/tazobactam regimen.

Table S2. Regression coefficients (Est.), p-values, adjusted odds ratios (adj. OR) with 95%-CIs from the multiple logistic regressions (Model A and Model B) including all covariates with ASMD ≥ 0.2.

Predictor	Est. (SE)	p	Adj. OR, 95%-CI
	Model A with fully observed covariates (n = 163)		
(Intercept)	-1.35 (0.51)	0.010	-
**Carbapenem vs. piperacillin/tazobactam**	**2.23 (0.48)**	**< 0.001**	**9.26 [3.78, 25.01]**
Male sex	0.15 (0.45)	0.740	1.16 [0.48, 2.87]
Implanted device[1]	-0.03 (0.55)	0.960	0.97 [0.33, 2.85]
Serratia	0.17 (0.50)	0.730	1.19 [0.44, 3.16]
Polymicrobial BSI	0.06 (0.52)	0.910	1.06 [0.38, 2.97]
Respiratory tract	-0.91 (0.58)	0.120	0.40 [0.12, 1.22]
Urinary tract	0.58 (0.58)	0.320	1.78 [0.57, 5.70]
Biliary tract	1.78 (0.66)	0.010	5.95 [1.69, 22.88]
Pitt bacteremia score	-0.47 (0.13)	< 0.001	0.63 [0.47, 0.78]
	Model B with incomplete covariates (n = 157)		
(Intercept)	-1.11 (0.54)	0.040	-
**Carbapenem vs. piperacillin/tazobactam**	**2.34 (0.52)**	**< 0.001**	**10.41 [3.97, 30.59]**
Male sex	0.33 (0.48)	0.490	1.39 [0.54, 3.64]
Implanted device[1]	-0.19 (0.58)	0.740	0.82 [0.26, 2.58]
Metastatic carcinoma/leukemia	-0.59 (0.84)	0.480	0.55 [0.09, 2.67]
Serratia	0.23 (0.52)	0.660	1.26 [0.45, 3.53]
Polymicrobial BSI	0.10 (0.55)	0.860	1.10 [0.37, 3.26]
Respiratory tract	-0.96 (0.61)	0.110	0.38 [0.11, 1.22]
Urinary tract	0.66 (0.67)	0.320	1.94 [0.53, 7.44]
Biliary tract	1.83 (0.69)	0.010	6.22 [1.66, 25.47]
Pitt bacteremia score	-0.29 (0.15)	0.050	0.75 [0.54, 0.98]
SOFA score at baseline	-0.14 (0.07)	0.050	0.87 [0.76, 1.00]

[1] Implanted devices include cardiac and orthopedic implanted devices.

References

1. Burgette, L.F.; McCaffray, D.F.; Griffin, B.A. Propensity score estimation with boosted regression. In: Pan W, Bai H, eds. Propensity Score Analysis: Fundamentals and Developments. New York (NY): *Guilford Press* **2015**, 49-73.
2. Onukwugha, E.; Bergtold, J.; Jain, R. A primer on marginal effects—Part I: Theory and formulae. *Pharmacoeconomics* **2015**, 33, 25-30.
